# Intraindividual Variability in Adolescent Impulsivity: The Predictive Role of Family and Peer Relationships

**DOI:** 10.1007/s10802-025-01340-y

**Published:** 2025-06-20

**Authors:** Gregory M. Fosco, Lan Chen, Jessica DeFelice

**Affiliations:** 1 Human Development and Family Studies, The Pennsylvania State University, University Park, PA 16802 USA; 2 The Pennsylvania State University, The Edna Bennett Pierce Prevention Research Center, University Park, PA 16802 USA

**Keywords:** Adolescent impulsivity, Family conflict, Parent-adolescent conflict, Peer rejection, Intensive longitudinal methods

## Abstract

Adolescent impulsivity is a robust risk factor for adolescent problem behaviors. Historically, impulsivity has been conceptualized as a trait characteristic; however, recent work conducted with adult samples indicates impulsivity also exhibits state-like qualities, fluctuating within persons from day to day. If this is also true for adolescents, it suggests that there are days of higher and lower risk for problem outcomes. This study was designed to (a) demonstrate that impulsivity exhibits meaningful within-person variability in adolescents as it does with adults, and (b) to evaluate family and peer relationship dynamics that may explain daily within-person changes in impulsivity. This study collected baseline surveys and 21 consecutive daily surveys from a sample of 135 parents and adolescents (54.8% female). Parents and adolescents reported on daily family cohesion and conflict and parent-adolescent closeness and conflict; adolescents reported on daily peer relationship satisfaction and rejection, and impulsivity. Multilevel models indicated that on days of elevated family conflict, parent-adolescent conflict, and peer rejection, adolescents experienced higher than usual impulsivity, especially in analyses of adolescent-reported data. In parent-report data, days of elevated parent-adolescent conflict (in the context of low parent-adolescent connectedness) also was associated with elevated adolescent impulsivity. These findings underscore the dynamic quality of adolescent impulsivity and indicate that interpersonal conflict may be a key day-level risk factor for elevated impulsivity.

**Supplementary Information:**

The online version contains supplementary material available at 10.1007/s10802-025-01340-y.

## Intraindividual Variability in Adolescent Impulsivity: Family and Peer Relationships Explain Daily Changes in Adolescent Impulsivity

Adolescent trait impulsivity is generally regarded as an individual risk factor for a host of problem outcomes. Trait impulsivity is generally defined as acting without forethought and can also include a tendency to accept immediate rewards rather than greater, but delayed rewards, difficulties with planning, and low self-control (van Baal, 2022; Beauchaine et al. 2017). This individual difference factor is recognized as an underlying risk for developmental trajectories of externalizing problems. Impulsivity is recognized as related to deficits in executive functioning, particularly inhibitory control (Leshem & Yefet, 2019) and plays a central role in models of developmental psychopathology, in which is serves as a heritable trait characteristic underlying risk for Attention-Deficit Hyperactivity Disorder (ADHD), Conduct Disorder, poor educational outcomes and problem peer association in adolescence, all of which ultimately set the stage for problematic substance use and antisocial behavior problems in adulthood (Beauchaine et al., 2009). A related model also places trait impulsivity as a key developmental risk factor for borderline personality disorder (Crowell et al., 2009). Trait impulsivity also has been linked with eating disorder risk (Kenny et al., 2019; Wonderlich et al. 2024) as well as non-suicidal self-injury (Riley et al., 2015). Thus, impulsivity is a risk factor of wide-reaching social significance.

Adding to the developmental psychopathology literature documenting a progression across disruptive behavior disorders from early life to adulthood, trait impulsivity also serves as a salient risk indicator at later developmental periods. For example, childhood trait impulsivity is associated with externalizing problems in later developmental periods (e.g., Stewart et al. 2023; Wang et al., 2016). Even in late adolescence, trait impulsivity signals risk for externalizing problems (Ahmad & Hinshaw, 2017) and addiction (Gullo et al. 2023). Thus, trait impulsivity is an important risk indicator across developmental periods.

## Intraindividual Variability in Impulsivity

Historically, impulsivity has been conceptualized as a trait characteristic, but recent evidence suggests that impulsivity also exhibits state-like qualities, fluctuating within-person on a day-to-day (or even moment-to-moment) basis. Studies of adult participants have revealed that state-level measures of impulsivity can reliably capture within-person variability (Tomko et al. 2014; Stevens et al., 2020). These studies disaggregating within- and between-person variability in impulsivity report intraclass correlations ranging from 0.35 to 0.44, indicating 56–65% of the variability in impulsivity may occur within-persons, rather than between-persons (Feil et al., 2020). In disentangling within-person from between-person variance, it is possible to capture both trait-like and state-like features of impulsivity. Trait-like conceptualizations reflect the dispositional aspect of impulsivity, consistent with studies assessing between-person differences in risk, or classification systems for diagnosing individuals with ADHD. State-like qualities refer to ways in which impulsivity changes within-person from day to day. Thus, individuals’ experiences of impulsivity may be more or less than their usual level. Simply stated: people have good and bad days. Because state impulsivity fluctuates within person, it may be malleable to situational factors such as interpersonal relationships, stress, or other experiences. Considering a state-like perspective of impulsivity enables novel insights into the factors that might help manage or exacerbate one’s tendency to be impulsive.

The emerging evidence regarding daily variation in impulsivity has important implications for individual well-being. A handful of studies now link within-person variation in impulsivity with daily mood. On days of higher impulsivity than usual, people tend to feel increased levels of negative affect (Stevens et al., 2020). Other work suggests that impulsivity may have lasting effects into the next day’s mood: days with higher than usual levels of impulsivity predict next-day diminished positive affect (Depp et al., 2016), and next-day elevated negative affect (Titone et al. 2022), although the link with negative affect is sometimes not evident (Depp et al., 2016). Nonetheless, daily variation in impulsivity appears to be important for daily mood and well-being.

State-level impulsivity is often also viewed as episodes of diminished inhibitory control (Chaku et al., 2024), underscoring how state impulsivity is linked to risks. For example, increases in state impulsivity may lead to increased *urges* to engage in problem behavior, such as the desire to engage in behaviors such as gambling, substance use, risky behavior, and eating (van Baal et al. 2022). Consistent with this work, a study using daily diary methods with a large sample of college students revealed that on days of high state impulsivity, participants reported more sexual risk-taking (Riley et al., 2019). Thus, state-level increases in impulsivity may serve as a signal for risk of engaging in serious problem behavior.

Daily variation in impulsivity may be a risk factor for substance use. For example, on days of elevated impulsivity, young adults were more likely to use alcohol (Griffin & Trull, 2021; Riley et al., 2019), with other work suggesting that days of high impulsivity may increase risk for heavy episodic drinking (Stamates et al., 2019; Stevens et al., 2020). At the same time, some studies fail to find a link between impulsivity and substance use either in the form of null findings in same-day analyses (e.g., Griffin et al., 2021; McGowan et al., 2022), other studies conducting lagged analyses have yielded results suggesting a reverse direction of effects in which substance use predicts elevated impulsivity on the following day (Stamates & Lau-Barraco, 2020). However, although findings regarding the link between state impulsivity and substance use are somewhat mixed at this early stage of research, they do suggest that daily variation in impulsivity may hold important implications for alcohol use risk.

To date, much of the work studying within-person variability in impulsivity has been focused on clinical populations (e.g., participants with borderline personality disorder; Tomko et al. 2014) and samples of late adolescents and adults (studies typically sampling participants that are 18 years or older). As a result, less is known about the degree to which impulsivity fluctuates for adolescents. However, if impulsivity does exhibit meaningful within-person fluctuations in adolescence, it would suggest that adolescents have days of higher and lower risk for problem behaviors. Understanding the factors that drive these within-person fluctuations could guide interventions around how to reduce short-term risk for adolescents’ distress, engagement in substance use, problem behavior, or even suicide.

## Understanding Day-Level Predictors of State Impulsivity

An important question remains: what are factors that explain day-to-day variability in adolescents’ impulsivity? By identifying daily experiences that elicit within-person changes in adolescent impulsivity, we may be better able to characterize the conditions under which adolescents are at elevated or decreased risk for problem behaviors. Further, by using within-person methods, we can identify malleable processes that may guide interventions aimed at reducing adolescent risk (Molenaar, 2004).

Little is known about the predictors of daily variation in adolescent impulsivity; to our knowledge, the work to date has also focused on late adolescents and adults (i.e., ages 18 and older). However, existing work suggests that negative affect and stressful experiences are important risks associated with variability in state impulsivity. In clinical and non-clinical samples, experiences of negative affect correspond to increases in impulsivity (Feil et al., 2020; Tomko et al., 2015); lagged analyses link elevated negative affect with increases in next-day impulsivity (Depp et al., 2016). Finally, a recent study evaluated daily stressors, positive mood, and daily mood as unique predictors of state impulsivity in a clinical sample. In this study (Sharpe et al., 2021), unique effects were found for each predictor: impulsivity was higher on days when negative affect was higher than usual, when participants experienced more stressors than usual, and when positive affect was lower than usual. Taken together, this literature suggests that identifying stressors in adolescents’ lives may be a fruitful avenue for identifying factors that account for daily variation in impulsivity.

Family and peer relations represent salient domains of adolescent daily experiences (Steinberg, 2001). The current study was designed to capture daily fluctuations in family, parent-adolescent, and peer relationships, and evaluate whether they explain within-person variability in impulsivity. As no studies have been conducted on this question to date, we turned to the developmental literature for preliminary evidence. Across family-level, parent-adolescent, and peer domains, longitudinal evidence suggests these interpersonal relations are associated with later impulsivity. For example, longitudinal panel model data indicate reciprocal relations between family conflict and trait impulsivity between late childhood (ages 9–10) and early adolescence (ages 11–12) (Yin et al., 2025). However, family conflict predicted late adolescent trait impulsivity, but reciprocal effects were not found during this period (Elam et al., 2016). Other work has found a longitudinal link between positive parent-adolescent relationship quality and later adolescent delay discounting (Kahn et al., 2015). Likewise, reciprocal relations between peer rejection and ADHD symptoms are evident in childhood (Stenseng et al., 2016), and again in adolescence (Stenseng et al., 2024) – a finding that was evident in early to middle adolescence (Ji et al., 2019). Thus, during the adolescent developmental period, there is evidence for prospective, reciprocal relations between impulsivity and interpersonal relationships; however, there seems to be a slight indication that in middle- to late-adolescence, interpersonal relations may be somewhat more of a driving factor in these processes.

## The Current Study

The current study was designed to address two research aims. First, we sought to replicate work documenting meaningful within-person variability in impulsivity among adults in a non-clinical sample of adolescents. Using a 21-daily diary design, we collected adolescent self-report data on their daily impulsivity. We expected to find that these daily reports of impulsivity exhibited meaningful within-person variability. We first tested this by calculating an intraclass correlation, and expected to find that 20% or more of the variance would be attributed to within-person variability, suggesting that we can evaluate predictors of daily variation in this construct (Bolger & Laurenceau, 2013).

Second, we evaluated family and peer predictors of daily variation in adolescent impulsivity. In the family domain, we examined family-level and parent-adolescent relationship quality. Specifically, we examined family-level cohesion and conflict, as well as parent-adolescent closeness and conflict. All four of these constructs have been found in prior research to (a) exhibit meaningful within-person variability, and (b) they each are linked with adolescent negative or positive affect (Bai et al., 2017; Chung et al., 2011; Coffey et al., 2020; Fosco et al., 2019, 2020; Fosco & Lydon-Staley, 2020; Lippold et al., 2016). Thus, we expected that, on days when family conflict and parent-adolescent conflict were higher than usual, adolescents would experience increased impulsivity. Conversely, we evaluated whether increases in family cohesion or parent-adolescent closeness would be associated with decreases in adolescent impulsivity.

In the peer domain, we considered peer rejection and peer relationship satisfaction as indicators of stressful (i.e., rejection) and pleasant (i.e., satisfaction) daily peer experiences. A robust literature documents that these aspects of peer relationships in adolescence exhibit meaningful day-to-day variability and also are linked with adolescents’ daily mood and well-being (Bai et al., 2017; Ha et al., 2019; Mayfield & Fosco, 2021; Morrow et al., 2014; Weinstein et al., 2006; Xu et al., 2023). Similar to analyses with family processes, we expected that experiences of peer rejection would correspond to increases adolescents’ impulsivity and days of high peer relationship satisfaction would correspond to decreases in adolescents’ impulsivity.

Because of the dearth of research studying daily family and peer factors in relation to adolescent impulsivity, we did not have specific hypotheses about which family or peer domains would have the most robust effects. Thus, we first examined these relations by domain (i.e., separate models for family-level cohesion and conflict, parent-adolescent closeness and conflict, and peer relationship satisfaction and rejection). We then used a model-building approach to test the unique effects of the significant predictors of adolescent impulsivity in an integrative model. Finally, given the evidence in the developmental literature for reciprocal relations, we conducted follow-up lagged analyses to test the direction of effects in our models.

## Method

### Participants

Participants, recruited through Pennsylvania high schools, were 135 parent-adolescent dyads. The inclusion criteria for participation were: (a) adolescent has been living with the caregiver for at least one year, (b) have daily access to the internet and ability to complete surveys online, (c) English fluency, (d) the target adolescent was in 9th, 10th, or 11th grade at the start of their participation, (e) both participating parent and adolescent consented to participate, (f) caregivers agree to allow their youth access on-line surveys when needed. This study was approved by the Penn State IRB (protocol #15632; Everyday Relationships in Adolescence).

Adolescent participants were 54.8% female (*n* = 74) and 45.2% male (*n* = 61) at birth (*M*_*age*_ = 15.58, *SD*_*age*_ = 1.17, range = 13–18). Adolescents identified their gender as Man (44.4%; *n* = 60), Women (50.4%; *n* = 68), Gender nonconforming (0.7%; *n* = 1), Non-binary (0.7%; *n* = 1), Trans male (0.7%; *n* = 1), or “something else describes me” (3.0%; *n* = 4). Adolescents identified their race as: White (85.9%; *n* = 116), African American/Black (13.3%; *n* = 18), Alaska Native or American Indian (0.7%; *n* = 1), Filipino (0.7%; *n* = 1), Asian Indian (2.2%; *n* = 3), Vietnamese (0.7%; *n* = 1), Korean (0.7%; *n* = 1), other Asian (0.7%; *n* = 1), and multiracial (5%; *n* = 7). Adolescents reported their ethnicity as: not of Hispanic, Latino, Latinx, or Spanish origin (92.6%; *n* = 125), Mexican, Mexican American, Chicano (1.5%; *n* = 2), Puerto Rican (2.2%; *n* = 3), and another Hispanic, Latino, Latinx, or Spanish origin (3.7%; *n* = 5).

Parents in this study identified their gender as: Woman (91.1%; *n* = 123), Man (7.4%; *n* = 10), Gender nonconforming (0.7%; *n* = 1), and decline to answer (0.7%; *n* = 1). Caregivers identified as the adolescents’ mother (92.6%; *n* = 125), father (6.7%; *n* = 9), or aunt (0.7%; *n* = 1). Parents identified their race as: White (87.4%; *n* = 118), African American/Black (12.6%; *n* = 17), Alaska Native/American Indian (0.7%; *n* = 1), Asian Indian (1.5%; *n* = 2), Vietnamese (0.7%; *n* = 1), Korean (0.7%; *n* = 1), Japanese (0.7%; *n* = 1), or “some other race” (1.5%; *n* = 2), or multiracial (5.9%; *n* = 8). Only one parent reported their ethnicity as Hispanic, Latino, Latinx, or Spanish origin (0.7%; *n* = 1). Parents marital status ranged from married (74.1%; *n* = 100), living together (3%; *n* = 4), separated (3.7%; *n* = 5), divorced (8.9%; *n* = 12), and single (10.4%; *n* = 14); most parents reported living with other adults (88.1%; *n* = 119). With a median income of $100,000-$124,999 per year, their annual household incomes ranging from less than $10,000 (2.2%) to $125,000 or more (47.4%). Parents’ education included high school graduate/GED certificate (4.4%; *n* = 6), Partial college (at least one year) or specialized training (5.9%; *n* = 8), Junior college/Associate’s degree (9.6%; *n* = 13), Standard college or University graduation (27.4%; *n* = 37), and Graduate professional training/graduate degree (52.6%; *n* = 71).

### Procedures

This study included baseline and 21-day daily diary protocol, sent to parents and adolescents. Families were recruited through emails or printed forms, which informed parents about the study and provided a link to a screening website, in collaboration with school partners. The school distributed informational flyers to the adolescents to make them aware of this study. After establishing family eligibility for the study, parents and their adolescents consented or assented to participate, and both completed baseline surveys. Then, participants met with project team member via Zoom video call for training in the daily diary surveys and to verify their identity. After this, parents and adolescents engaged sequence of 21 consecutive daily surveys were individually delivered to parents and adolescents via text message or email (depending on their preference). Surveys were sent at 6:00 am, with text-message reminders sent between 10:00 am and 12:00 pm. Participants were asked to report on their experiences from the previous day and instructed to complete surveys privately and independently. Daily survey compliance was high. Parents completed 93.4% ($$\:{M}_{Parent}$$ = 19.61, $$\:S{D}_{Parent}$$ = 3.53) and adolescents completed 86.1% ($$\:{M}_{Adolecsent}$$ = 18.08, $$\:S{D}_{Adolecsent}$$ = 4.65) of daily surveys. Given the high compliance, all participants were analyzed for all days with available data (days with missing data were dropped from analysis). Participants were compensated with $25 for completing baseline surveys and up to $25 per week of daily surveys.

## Measures

### Daily Adolescent Impulsivity

Each day, adolescents rated two impulsivity items, adapted from the Momentary Impulsivity Scale (Tomko et al. 2014). The original scale is a four-item measure developed with adult samples. We selected two items from this scale, “How much of the time yesterday did you say things without thinking?” “Did you make ‘spur of the moment’ decisions?”. We omitted “I spent more money than I meant to” so that our scale would be relevant to youth from high-income and low-income families; additionally, we omitted the item “I felt impatient” which has been identified as having a poor factor loading in prior research (Stevens et al., 2020). We evaluated the daily scale to determine their reliability in capturing within-person variability (R_c_; Bolger & Laurenceau, 2013) and between-person reliability, accounting for repeated measures (R_1F_; Cranford et al., 2006). In this sample, the scale demonstrated acceptable within-person and between-person reliabilities ($\:{R}_{1F}$= 0.52, $\:{R}_{c}$= 0.77).

### Daily Family-Level Cohesion and Conflict

Each day, parents and adolescents each were asked to rate how family members got along with each other the prior day (before going to bed). Participants rated two items measuring *family cohesion*, including “Family members really helped and supported one another,” “There was a feeling of togetherness in our family.” They also rated two items measuring *family conflict* “Family members criticized one another,” and “Family members fought.” Daily family cohesion and conflict scores were calculated separately for adolescents and parents by averaging the respective items. Family cohesion and conflict were both adapted from the shortened Family Environment Scale (Bloom, 1985) for daily data collection. The scores ranged from 0 (not at all) to 10 (a lot), in increments of 0.1. A higher score indicates greater family cohesion or conflict. Family cohesion demonstrated good within- and between-person reliabilities (adolescent/parent: $\:{R}_{1F}\:$= 0.76/0.79, $\:{R}_{c}$= 0.90/0.84). Family conflict demonstrated good within- and between-person reliabilities (adolescent/parent: $\:{R}_{1F}$= 0.77/0.80, $\:{R}_{c}$= 0.73/0.67).

### Daily Parent-Adolescent Connectedness and Conflict

Each day, parents and adolescents were asked to report on their relationship with each other, regarding the prior day. Parents and adolescents each rated their perceptions of connectedness with the other. *Parent-adolescent connectedness* was assessed using an average of two items, “How close and connected did you feel to your [caregiver]/ [child] yesterday” and “How much did you feel loved by your [caregiver]/ [child] yesterday”. *Parent-adolescent conflict* items differed slightly across reporters. Adolescent items included: “How angry or mad was your [caregiver] with you,” and “How much tension was there between you and your [caregiver]”. Parents items included: “I was angry or mad at [child]” and “There was tension between [child] and you.”

For youth items, relationships were piped into the items to reflect the participating caregivers (e.g., “mother”, “father”). The participating child’s name was piped into the questions for caregivers. All items were rated using a 0 to 10 (in 0.1 increments) slider. Connectedness demonstrated acceptable within- and between-person reliabilities (adolescent/parent: $\:{R}_{1F}$= 0.68/0.75, $\:{R}_{c}$= 0.87/0.87). Conflict demonstrated acceptable within- and between-person reliabilities (adolescent/parent: $\:{R}_{1F}$= 0.79/0.80, $\:{R}_{c}$= 0.73/0.63).

### Daily Peer Relationship Satisfaction and Rejection

Adolescents were asked about their friendships during the prior day. *Peer satisfaction* was measured using two items from the Investment Model Scale (Branje et al., 2007): “I felt satisfied with my friendship(s),” and “I felt close and connected to my friend(s).” Adolescents reported on their daily *peer rejection* using a two-item measure, “I argued or fought with my friend(s)” and “I felt rejected by my friend(s).” Each was answered using a 0–10 slider, with 0.1 increments, respective items were averaged and scored so that higher values reflect more satisfaction or rejection. Peer satisfaction ($\:{R}_{1F}$= 0.81, $\:{R}_{c}$= 0.88) and peer rejection ($\:{R}_{1F}$= 0.51, $\:{R}_{c}$= 0.57) exhibited acceptable within- and between-person reliabilities.

### Analytic Plan

To evaluate Hypothesis 1, whether there is meaningful daily variability in adolescent impulsivity, we conducted a variance decomposition by fitting a means model with a random intercept, wherein participant ID predicted daily adolescent impulsivity. This model partitioned the total variance in daily adolescent impulsivity into between-person and within-person components. The intraclass correlation coefficient (ICC) was calculated as: $\:\text{ICC}=\frac{{{\upsigma\:}}_{\text{between}}^{2}}{{{\upsigma\:}}_{\text{between}}^{2}+{{\upsigma\:}}_{\text{within}}^{2}}$.

For Hypothesis 2, we conducted multiple multilevel models to capture the daily adolescent impulsivity because it is particularly well-suited for addressing the inherent nested nature of intensive repeated measures - specifically, 21 days of observations nested within individuals (Snijders & Bosker, 2011). Before computing the models, we calculated each person’s average level of the variables across all study days to get between-person level variables, which were grand-mean centered (between-person effects). We then used person-mean centering approaches to compute the within-person levels for all daily variables, which represents the deviation of a certain day’s score from the person’s average level across all study days. Positive values of within-person variables reflect days in which values were higher than average; negative values reflect levels that are lower than usual for a particular person.

We organized independent variables into groups: family-level relationships (i.e., cohesion and conflict), parent-adolescent relationships (i.e., connectedness and conflict), and peer relationships (i.e., satisfaction and rejection). To evaluate these domains, we conducted a set of models: Models 1–4 tested family-level and parent-adolescent relationships with both adolescent and parent reports, and Model 5 tested adolescent-reported peer relationships. Each model evaluated the main effects and interaction effects of the respective variables (see Supplemental Fig. 1 for a prototypical model equation). When significant interaction effects emerged, we used Johnson-Neyman analysis to identify the range of significant (or nonsignificant) interaction effects (Johnson & Fay, 1950). Subsequently, we synthesized the findings into comprehensive models – one for adolescent-report (Model 6) and one for parent-report (Model 7) data – by including significant predictors from the earlier models. All models included time (centered at the midpoint of the daily surveys; Bolger & Laurenceau, 2013), between-person levels of predictors, adolescent age, adolescent sex (0 = female, 1 = male), and family incomes as covariates. All models included random intercepts and random slopes for day-level predictors, allowing both the average level of impulsivity and the strength of within-person associations to vary across individuals.

Due to the moderate sample size and complex mixed-effect models with extensive variance-covariance parameters, we met singularity issues in the parent-reported adolescent relationship model (Model 4) and final models (Model 6 and 7). Singularity occurs when certain dimensions of the variance-covariance matrix become redundant, resulting in zero estimates for variance components or extremely high correlation coefficients (Bates et al., 2015; Barr et al., 2013). To facilitate model convergence and mitigate singularity issues, we conducted a principal component analysis (PCA) on the random effects and fixed certain variables. The PCA identified a principal component with a near-zero standard deviation, indicating that one of the random effects had very little variability and was likely redundant. The PCA rotation matrix revealed the variable most highly correlated with this near-zero component, suggesting that its random effect was contributing minimally to the model and leading to singularity. Specifically, we fixed the random effect for parent-reported parent-adolescent connectedness in Model 4, which showed the lowest variability in this model. Similarly, we fixed adolescent-reported parent-adolescent conflict in Model 6 and parent-reported parent-adolescent connectedness in Model 7 to ensure more robust model performance. Analyses were conducted in R Studio (version 4.3.2) using the lmer function in the lme4 package (Bates et al., 2015).

### Post-Hoc Analysis

Because prior tests focused on same-day relations between family and peer relations and impulsivity, we conducted follow-up multilevel models to test whether direction of effects might be detected in these results. First, we tested prospective paths between prior-day family and peer relations as predictors of next-day adolescent impulsivity. Second, we reversed the direction, testing prior-day impulsivity as a predictor of each of the family and peer outcomes.

## Results

**Fig. 1:**
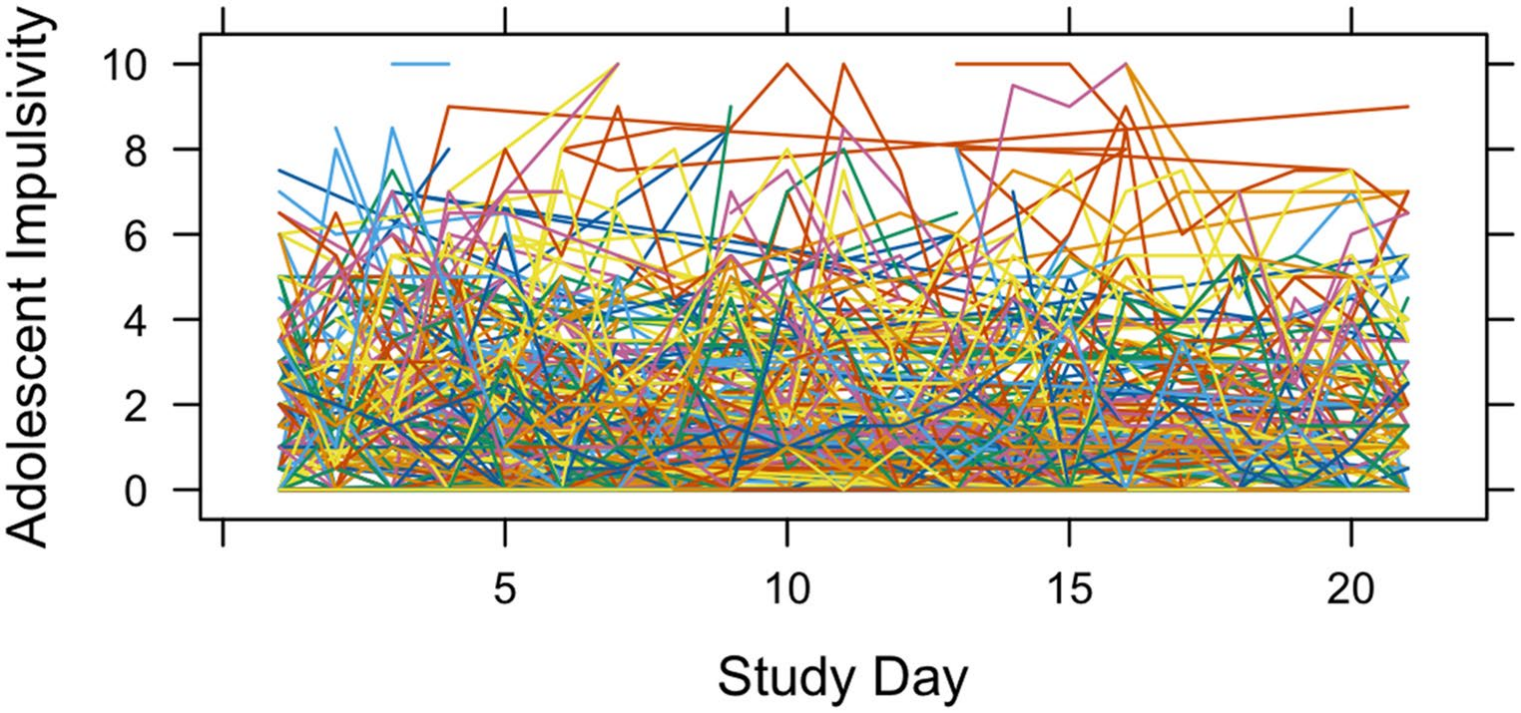
The individual trajectories of adolescent impulsivity across 21 study days

Table 1 presents the descriptive statistics and correlations for the study variables at both within-person and between-person levels; demographic data are provided in Supplemental Table S1. To address our first research question, we conducted a variance decomposition analysis to examine the within-person variability in adolescent impulsivity. The ICC was 0.6018, indicating that 60.18% of the daily variability in adolescent impulsivity can be attributed to between-person differences and 39.82% can be attributed to within-person variability. Based on guidance from Bolger and Laurenceau (2013), this surpasses the acceptable level (20%) of within-person variability needed to proceed with analyses of within-person process. Thus, our hypothesis that there would be meaningful within-person variability was supported. An illustrative plot of daily variation in adolescent impulsivity of the sample is presented in Fig. 1.

**Table 1:** Correlations and intraclass correlation coefficient

	1	2	3	4	5	6	7	8	9	10	11
1. Impulsivity	--	-0.18**	0.20**	-0.01	0.11**	-0.13**	0.29**	-0.04*	0.27**	-0.06**	0.32**
2. Family Cohesion A	-0.06*	--	-0.39**	0.27**	-0.21**	0.81**	-0.37**	0.28**	-0.28**	0.47**	-0.26**
3. Family Conflict A	0.12**	-0.27**	--	-0.29**	0.38**	-0.36**	0.71**	-0.25**	0.32**	-0.34**	0.56**
4. Family Cohesion P	-0.01	0.19**	-0.19**	--	-0.40**	0.27**	-0.21**	0.71**	-0.34**	0.14**	-0.02
5. Family Conflict P	0.02	-0.12**	0.36**	-0.34**	--	-0.25**	0.30**	-0.29**	0.61**	0.00	0.01
6. PC Connectedness A	-0.03	0.38**	-0.23**	0.20**	-0.11**	--	-0.43**	0.41**	-0.40**	0.43**	-0.25**
7. PC Conflict A	0.13**	-0.25**	0.52**	-0.16**	0.32**	-0.30**	--	-0.32**	0.57**	-0.30**	0.52**
8. PC Connectedness P	-0.01	0.19**	-0.17**	0.50**	-0.22**	0.31**	-0.24**	--	-0.51**	0.06**	-0.05*
9. PC Conflict P	0.05*	-0.14**	0.34**	-0.23**	0.49**	-0.17**	0.47**	-0.33**	--	0.02	0.08**
10. Peer Satisfaction	0.01	0.03	-0.03	-0.00	-0.04	0.09**	-0.05*	0.03	-0.03	--	-0.46**
11. Peer Rejection	0.13**	-0.09**	0.07*	-0.04*	0.02	-0.05*	0.11**	-0.04	0.02	-0.18**	--
ICC	60.18%	65.39%	36.87%	51.26%	27.56%	67.19%	33.86%	60.19%	23.80%	55.63%	38.27%
Mean	2.10	6.85	1.82	7.33	1.41	6.84	1.41	7.35	1.12	7.76	0.76
SD	1.95	2.22	2.24	2.01	1.89	2.36	2.01	2.30	1.94	2.31	1.34

Note: A = Adolescent-report, P = Parent-report, PC = Parent-child, ICC = Intraclass correlation coefficient, SD = Standard deviation, ** *p* <.01, * *p* <.05. Between-person (average) correlations are shown above the diagonal; within-person (daily) correlations are shown below the diagonal

In the next step, we computed three sets of multilevel models separately to evaluate whether variability in family-level, parent-adolescent, and peer relations corresponded to changes in adolescents’ state impulsivity. Within each domain, we evaluated main effects and interactive effects. If interactions were not statistically significant, they were omitted from the final (presented) model for parsimony. Models evaluating family-level and parent-adolescent relations, models were computed separately for adolescent-report and parent-reports of family relations. Parents did not report on peer relationships, so only one set of analyses was conducted on peer relationships. Thus, five multilevel models were computed for the first set of analyses.

Table 2 reports the findings from the analyses of family-level and parent-adolescent relationships. In three of the four models (Models 1–3), no significant interaction effects were found; thus, only the main effects are reported. We first examined the models examining family-level conflict and cohesion in relation to adolescent impulsivity. In the adolescent-report model (Model 1), family conflict was a risk, such that on days of higher family conflict, adolescents reported increases in their impulsivity ($\:\beta\:$ = 0.11, *p* <.01); no relation was found for family cohesion. In the model evaluating parent-reported family processes, neither family conflict nor family cohesion were associated with adolescent impulsivity (Model 2).

**Table 2:** Family-level and parent-adolescent relationship predictors of impulsivity

	Model 1 Youth-report		Model 2 Parent-report	Model 3 Youth-report	Model 4 Parent-report
	Est/Var (SE)	Est/Var (SE)		Est/Var (SE)	Est/Var (SE)
Intercept	2.71 (2.13)	3.20 (2.11)		2.46 (2.15)	2.98 (2.16)
*Within-Person Fixed Effects*					
Daily Fam. Cohesion	-0.05 (0.03)	-0.01 (0.03)		----	----
Daily Fam. Conflict	0.11 (0.04) **	0.01 (0.03)		----	----
Daily Fam. Cohesion*Conflict	(ns)	(ns)		----	----
Daily PC Connected	----	----		0.00 (0.03)	0.00 (0.02)
Daily PC Conflict	----	----		0.12 (0.03) **	0.02 (0.03)
Daily PC Connected*Conflict	----	----		(ns)	-0.03 (0.01) **
Time	-0.02 (0.00) **	-0.02 (0.00) **		-0.02 (0.00) **	-0.02 (0.00) **
*Between-Person Fixed Effects*					
Avg. Fam. Cohesion	-0.12 (0.08)	0.05 (0.10)		----	----
Avg. Fam. Conflict	0.26 (0.15)	0.15 (0.18)		----	----
Avg. PC Connected	----	----		-0.01 (0.08)	0.09 (0.08)
Avg. PC Conflict	----	----		0.41 (0.15) **	0.51 (0.19) **
Youth Age	-0.02 (0.13)	-0.03 (0.13)		-0.02 (0.13)	-0.04 (0.13)
Youth Sex	0.33 (0.31)	0.10 (0.30)		0.30 (0.31)	0.15 (0.31)
Family Income	-0.06 (0.05)	-0.08 (0.05)		-0.03 (0.05)	-0.05 (0.05)
*Random Effects*					
Intercept 𝜎	2.73 (1.65)	2.89 (1.70)		2.78 (1.67)	2.75 (1.66)
Daily Fam. Cohesion	0.05 (0.23)	0.02 (0.12)		----	----
Daily Fam. Conflict	0.03 (0.19)	0.02 (0.14)		----	----
Daily PC Connectedness	----	----		0.04 (0.19)	(fixed)
Daily PC Conflict	----	----		0.03 (0.16)	0.02 (0.12)

Note: PC = Parent-child, Avg. = Average, ns = non-significant. ** *p* <.01, * *p* <.05

**Fig. 2:**
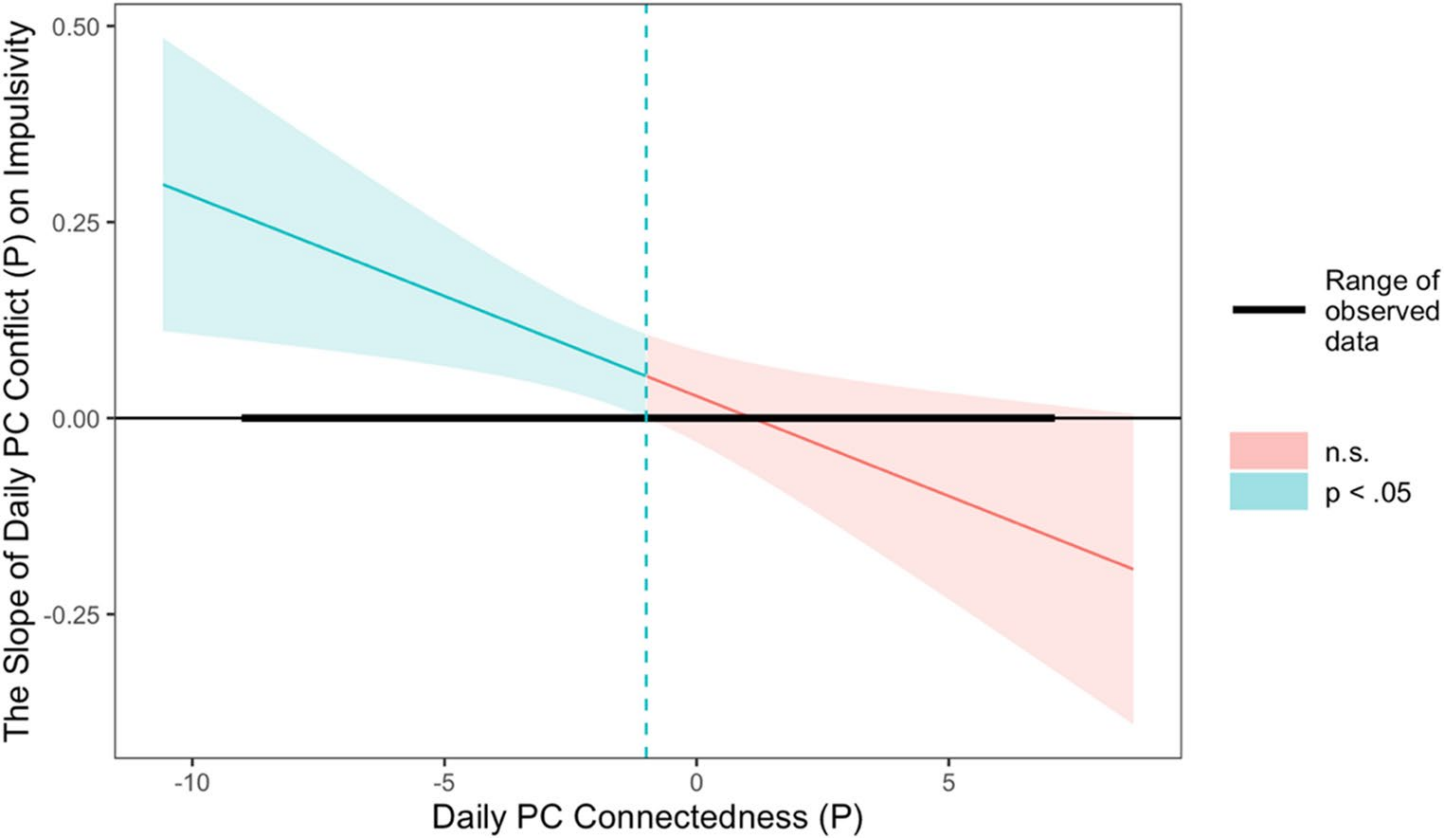
Johnson-Neyman plots of parent-report PC connectedness and PC conflict on adolescents’ impulsivity in Model 4

In the models examining parent-adolescent relationships, adolescent report of parent-adolescent conflict was associated with impulsivity, but parent-adolescent connectedness was not (Model 3). Specifically, on days when adolescents perceived higher than usual levels of conflict with their parents, they experienced increases in their impulsivity ($\:\beta\:$ = 0.12, *p* <.01). In the model evaluating parent-reported data (Model 4), there was a statistically significant interaction between parent-adolescent conflict and connectedness ($\:\beta\:$ = − 0.03, *p* <.01). To probe this interaction, we conducted a Johnson-Neyman analysis, which indicated that on days when parent-adolescent conflict was higher than usual, adolescents reported higher levels of impulsivity, but only when parent-adolescent connectedness was low (See Fig. 2).

We then computed a model of peer rejection and peer relationship satisfaction in relation to daily impulsivity (Table 3). Peer relationship satisfaction was not associated with impulsivity; however, on days when peer rejection was higher than usual, adolescents reported increased impulsivity ($\:\beta\:$ = 0.19, *p* <.01). A similar pattern emerged in the between-person effects: average peer rejection was positively associated with daily adolescent impulsivity ($\:\beta\:$ = 0.68, *p* <.01), but no relation was found regarding satisfaction with peer relationships.

**Table 3:** Peer relationship predictors of impulsivity

	Model 5
	Est/Var (SE)
Intercept	3.18 (2.04)
*Within-Person Fixed Effects*	
Daily Peer Satis.	0.03 (0.03)
Daily Peer Reject.	0.19 (0.05) **
Daily Peer Satis.*Reject.	(ns)
Time	-0.02 (0.00) **
*Between-Person Fixed Effects*	
Avg. Peer Satis.	0.06 (0.10)
Avg. Peer Reject.	0.68 (0.19) **
Youth Age	-0.07 (0.13)
Youth Sex	0.47 (0.29)
Family Income	-0.03 (0.05)
*Random Effects*	
Intercept 𝜎	2.61 (1.62)
Daily Peer Satis.	0.02 (0.15)
Daily Peer Reject.	0.06 (0.25)

Note: Avg. = Average, ** *p* <.01, * *p* <.05

In the final step, we integrated all statistically significant predictors of impulsivity into a comprehensive model (See Table 4) and conducted the analyses separately for adolescent-reported family processes and parent-reported family processes. The adolescent-report model (Model 6) largely replicated findings from the separate models described above. On days when adolescents perceived higher family conflict ($\:\beta\:$ = 0.09, *p* <.05), parent-adolescent conflict ($\:\beta\:$ = 0.05, *p* <.05), and peer rejection ($\:\beta\:$ = 0.16, *p* <.01) than usual, they reported increases in impulsivity. Regarding between-person effects, average parent-adolescent conflict ($\:\beta\:$ = 0.41, *p* <.05) and peer rejection ($\:\beta\:$ = 0.51, *p* <.05) were positively associated with adolescent impulsivity. The parent-report model (Model 7) also replicated results from models testing domains separately, described above. Indeed, the interaction between parent-adolescent conflict and connectedness was observed again ($\:\beta\:$ = − 0.03, *p* <.01), as was the effect for daily peer rejection ($\:\beta\:$ = 0.14, *p* <.01), average peer rejection ($\:\beta\:$ = 0.64, *p* <.01), and average parent-adolescent conflict ($\:\beta\:$ = 0.44, *p* <.05) positively predicted daily impulsivity.

**Table 4:** Family-level, Parent-Adolescent, and peer relationship predictors of impulsivity

	Model 6 Youth model	Model 7 Parent model
	Est/Var (SE)	Est/Var (SE)
Intercept	2.79 (2.07)	2.99 (2.05)
*Within-Person Fixed Effects*		
Daily Family Conflict (A)	0.09 (0.04) *	----
Daily PC Conflict (A)	0.05 (0.03) *	----
Daily PC Connectedness (P)	----	0.01 (0.02)
Daily PC Conflict (P)	----	0.01 (0.03)
Daily PC Connectedness*Conflict (P)	----	-0.03 (0.01) **
Daily Peer Rejection (A)	0.16 (0.04) **	0.14 (0.04) **
Time	-0.02 (0.00) **	-0.02 (0.00) **
*Between-Person Fixed Effects*		
Avg. Family Conflict (A)	-0.25 (0.21)	----
Avg. PC Conflict (A)	0.41 (0.19) *	----
Avg. PC Connectedness (P)	----	0.08 (0.08)
Avg. PC Conflict (P)	----	0.44 (0.18) *
Avg. Peer Rejection (A)	0.51 (0.20) *	0.64 (0.17) **
Youth Age	-0.04 (0.13)	-0.06 (0.13)
Youth Sex	0.18 (0.29)	0.19 (0.29)
Family Income	----	-0.03 (0.05)
*Random Effects*		
Intercept 𝜎	2.57 (1.60)	2.49 (1.58)
Daily Family Conflict (A)	0.04 (0.21)	----
Daily PC Conflict (A)	0.01 (0.08)	----
Daily PC Conflict (P)	----	0.02 (0.13)
Daily PC Connectedness (P)	----	(fixed)
Daily Peer Rejection (A)	0.03 (0.17)	0.02 (0.16)

Note: A = Adolescent-report, P = Parent-report, PC = Parent-child, Avg. = Average, **p <.01, *p <.05

Random intercepts and slopes were also estimated for day-level variables. The random intercept captured individual differences in adolescents’ average levels of impulsivity. Across models, the random intercept variances (e.g., $\:{\sigma\:}^{2}$=2.49 to $\:{\sigma\:}^{2}$ =2.89) indicated significant individual differences in adolescents’ average levels of impulsivity. Random slopes reflect individual differences in the magnitude of within-person associations observed in the day-level predictors and impulsivity. In other words, it allowed the effect of daily experiences on impulsivity to vary across adolescents. For example, in Model 1 (Table 2), the random slope variances for daily family cohesion ($\:{\sigma\:}^{2}$=0.05) and family conflict ($\:{\sigma\:}^{2}$=0.03) suggested that adolescents varied in the association between daily family relationship and impulsivity.

### Post-Hoc Analyses Testing the Direction of Effects

Follow-up analyses were conducted to evaluate the prior-day effects of family-level, parent-adolescent, and peer relationships on adolescent impulsivity. As shown in Supplemental Table S2, when adolescents reported higher family conflict than usual on the prior day, they reported significantly higher impulsivity ($\:\beta\:$ = 0.06, *p* <.05). Additionally, when prior-day adolescent-reported parent-adolescent conflict ($\:\beta\:$ = 0.06, *p* =.078) or peer rejection ($\:\beta\:$ = 0.06, *p* =.060) was higher than usual, adolescents reported marginally higher impulsivity. Next, we tested the prior-day effects of adolescent impulsivity on these predictors. As shown in Supplemental Table S3, prior-day adolescent impulsivity was not significantly associated with adolescent-reported family conflict, parent-adolescent conflict, or peer rejection.

## Discussion

Adolescent trait impulsivity has long been recognized as an underlying risk characteristic in ADHD, Conduct Disorder, Substance Use Disorder, and Antisocial Personality Disorder (Beauchaine et al., 2009), while other work links trait impulsivity to borderline personality disorder (Crowell et al., 2009), eating disorders (Kenny et al., 2019; Wonderlich et al., 2024) and non-suicidal self-injury (Riley et al., 2015). Recently, we have begun to expand our notion of impulsivity beyond conceptualizing it as a trait characteristic, to consider within-person fluctuations in state impulsivity. The current study had two goals. First, we sought to replicate prior work studying adults to determine whether there is meaningful within-person variability in impulsivity among adolescents. Second, we sought to identify family and peer processes that contribute to adolescents’ fluctuations in impulsivity. Ultimately, by examining daily processes underlying within-person variability in impulsivity, we hoped to better understand the conditions under which adolescents are at more or less at risk for poorer mental health or risky behaviors.

### Adolescent Impulsivity Exhibits both State and Trait Qualities

Regarding the first research question, we found that nearly 40% of the variance in adolescent impulsivity occurred at the within-person level, which exceeds conventional minimum criteria of 20% as a meaningful proportion of variance (Bolger & Laurenceau, 2013). This supports the premise that adolescent impulsivity has both state and trait qualities, consistent with research conducted with adult samples (typically ages 18 and older). Thus, our findings converge with the growing number of studies of adults, across clinical and non-clinical samples, and across measures of impulsivity, document that there is reliable within-person change in state impulsivity across occasions (e.g., days or moments). Although several studies of adult state impulsivity do not report intraclass correlations, those that do suggest that adult impulsivity may have even greater variance at the within-person level, with estimates of within-person variance exceeding 50% (Feil et al., 2020). Thus, it seems that adolescent impulsivity may exhibit comparable degrees of within-person variability to estimates from studies of adults.

By conceptualizing adolescent impulsivity as comprised of both state and trait qualities, it is now possible to construct a more nuanced perspective on impulsivity during this developmental period that has such long-lasting implications for life course development and developmental psychopathology. Our data reveal meaningful within-person variability in adolescent impulsivity, suggesting that adolescents have days of higher and lower risk for problem behaviors. This is an important first step in the study of state impulsivity, but additional work is needed. For example, it would be helpful to better understand the timescale at which impulsivity fluctuates – possibly changing in momentary time, or across hours or longer periods of the day. In addition, it will be helpful to know the typical timeframe for impulsivity to return to typical levels. Such work would provide a more granular understanding of how impulsivity fluctuates within adolescents.

### Family and Peer Predictors of Adolescent State Impulsivity

The second goal of this study was to evaluate family and peer relationship predictors of within-person changes in adolescent impulsivity. Our study benefitted from both parent and adolescent report of family-level relations and parent-adolescent relationship quality; thus, models were computed twice to evaluate how robust findings were across reporters. At the family level, in the adolescent-report data, daily family conflict, but not family cohesion corresponded to changes in adolescent state impulsivity (even in the lagged analyses). However, this finding was not replicated in the parent-report model. Thus, it may be that on days when adolescents perceived family conflict to be higher than usual, adolescents experienced elevated state impulsivity. The differences in findings by reporter raise questions about how best to interpret these findings. On one hand, it is common for parents and adolescents to have different perceptions of family functioning (de Los Reyes, 2011; McCauley et al., 2021). Guided by this premise, it may be that adolescents’ subjective perceptions of family conflict play a more salient role in predicting their state impulsivity. However, an alternative perspective is that family conflict may be a less robust predictor of impulsivity, evidenced in our failure to detect an effect when cross-reporter analyses were conducted. Across both parent- and adolescent-report models, family cohesion was not associated with variability in adolescent state impulsivity.

Findings for parent-adolescent relationship quality were more robust across reporters than findings for family-level constructs. Across both sets of models, on days when parent-adolescent conflict was higher than usual, adolescents experienced elevated state impulsivity. This finding was especially true in the adolescent-report model, evidenced by a significant main effect; however, in the parent-report model, the relation between parent-adolescent conflict and impulsivity was moderated by parent-adolescent connectedness. That is, days of higher parent-adolescent conflict corresponded to elevated adolescent impulsivity, when parent-adolescent closeness was lower than usual. In the parent model, the relation between parent-adolescent conflict and adolescent impulsiveness was not evident when parent-adolescent connectedness was higher. This pattern of results across both parent and adolescent models suggests that parent-adolescent conflict is a relatively robust predictor of state impulsivity. It is possible that the moderating effect of parent-adolescent connectedness reflects a degree of conflict intensity. That is, it may be that from a parents’ perspective, the parent-adolescent relationship may experience ruptures – characterized by higher levels of conflict and lower levels of connectedness – that impact adolescent state impulsivity.

Regarding peer relationships, our findings suggest that days in which adolescents feel more rejected by peers than usual confer risk for elevated state impulsivity. However, these findings were not evident in relation to peer relationship satisfaction. Thus, it seems that conflict with peers may be the more salient factor for adolescent risk for increased state impulsivity. However, these findings underscore the importance of supporting adolescents in coping with peer rejection effectively.

Together, the findings from this study highlight how daily interpersonal relations can dampen or exacerbate one’s general tendency toward impulsivity. Specifically, our results yielded a relatively consistent pattern of findings supporting conflict and rejection as key risk processes for elevated states of impulsivity, whereas positive relationship processes – family cohesion, parent-adolescent connectedness, or peer relationship satisfaction – were consistently not linked to within-person changes in impulsivity. Thus, our findings suggest that conflict is a key factor for impulsivity, while positive relationship quality may be less salient for adolescent state impulsivity. These findings are consistent with adult studies that link daily stressors and negative affect with increases in state impulsivity (Depp et al., 2016; Feil et al., 2020; Sharpe et al., 2021; Tomko et al., 2015). Interestingly, although some findings in studies of adults find a link between positive affect and impulsivity (e.g., Sharpe et al., 2021), other work using lagged analyses suggest a direction of effects in which impulsivity predicts later positive affect, but positive affect does not predict later impulsivity. Thus, it may be that negative affect and stressful experiences are key risks for adolescent impulsivity and positive relational experiences are not.

Our findings linking daily family conflicts and peer rejection with elevations in state impulsivity point to opportunities for targeted preventive interventions. One opportunity is to identify ways to structure daily experiences that minimize experiences of conflict before youth engage in contexts where impulsivity may undermine their success. For example, parents might structure morning routines to avoid risk for conflicts before school to support adolescent’s academic success. Similarly, interventions focused on promoting effective conflict resolution in family and peer relationships may help reduce the impact of interpersonal conflicts on vulnerable adolescents for whom elevations from their trait-levels of impulsivity may put them at risk. Future research is needed to investigate factors that might moderate these effects (e.g., conflict resolution may buffer the effects of conflict on impulsivity), and underlying mechanisms linking interpersonal conflict and rejection with state-level impulsivity.

### Limitations and Future Directions

Interpretation of the current findings should be tempered within the limitations of the study. First, this study was primarily comprised of White participants of higher socioeconomic status. Replication of these findings with more racially and socioeconomically diverse samples would be useful. In addition, it would be helpful to replicate these analyses with a clinical sample of youth who are high in trait impulsivity to see how these processes unfold. Our measure of impulsivity was most consistent with the concept of “rash” impulsivity, referring specifically to actions without forethought and planning. However, other measures, providing a more granular assessment of impulsivity domains may help shed more light on the relations between interpersonal relationship quality and adolescent state impulsivity. Although our study provides a useful first step in documenting daily-timescale processes, future research collecting data on multiple occasions each day would offer more insights into momentary impulsivity processes and help illuminate the timescale of impulsivity dynamics.

## Conclusions

Adolescent trait impulsivity is a robust risk indicator for a wide range of psychopathology outcomes. Our study documents that adolescent impulsivity exhibits both trait-like and state-like qualities, calling for an expanded conceptualization of adolescent impulsivity. In addition, our findings suggest that conflict in interpersonal relationships – family-level, parent-adolescent, and peer – appear to be key risk factors for change in adolescent state impulsivity. These findings suggest that interventions may fruitfully target conflict in close relationships to reduce adolescent risk for impulsivity.

## Electronic Supplementary Material

Below is the link to the electronic supplementary material.

Supplementary Material 1

## Data Availability

Data are available upon reasonable request to the corresponding author.
