# Supplementary material for: Intraindividual Variability in Adolescent Impulsivity: The Predictive Role of Family and Peer Relationships
Source: Res Child Adolesc Psychopathol. 2025 Jun 20;53(9):1367–80. doi: 10.1007/s10802-025-01340-y (PMC12423169; doi:10.1007/s10802-025-01340-y)
Supplement: Supplementary file 1 — Supplementary Material 1 [file 10802_2025_1340_MOESM1_ESM.docx]

**Table S1**. *Demographic Characteristics of Parent–Adolescent Dyads (N = 135)*

| **Variable** | **n** | **%** |  |
| --- | --- | --- | --- |
| **Adolescents** |  |  |  |
| *Sex Assigned at Birth* |  |  |  |
| Female | 74 | 54.8 |  |
| Male | 61 | 45.2 |  |
| *Gender Identity* |  |  |  |
| Woman | 68 | 50.4 |  |
| Man | 60 | 44.4 |  |
| Gender nonconforming | 1 | 0.7 |  |
| Non-binary | 1 | 0.7 |  |
| Trans male | 1 | 0.7 |  |
| Something else describes me | 4 | 3.0 |  |
| *Race* |  |  |  |
| White | 116 | 85.9 |  |
| African American/Black | 18 | 13.3 |  |
| Alaska Native/American Indian | 1 | 0.7 |  |
| Filipino | 1 | 0.7 |  |
| Asian Indian | 3 | 2.2 |  |
| Vietnamese | 1 | 0.7 |  |
| Korean | 1 | 0.7 |  |
| Other Asian | 1 | 0.7 |  |
| Multiracial | 7 | 5.2 |  |
| *Ethnicity* |  |  |  |
| Not Hispanic/Latino/Latinx/Spanish | 125 | 92.6 |  |
| Mexican/Mexican American/Chicano | 2 | 1.5 |  |
| Puerto Rican | 3 | 2.2 |  |
| Hispanic/Latino/Latinx/Spanish | 5 | 3.7 |  |
| *Age* |  |  |  |
| Mean (SD) | 15.58 (1.17) | -- |  |
| Range | 13–18 | -- |  |
| **Parents** |  |  |  |
| *Sex Assigned at Birth* | |  |  |
| Female | | 124 | 91.4 |
| Male | | 11 | 8.1 |
| *Gender Identity* |  |  |  |
| Woman | 123 | 91.1 |  |
| Man | 10 | 7.4 |  |
| Gender nonconforming | 1 | 0.7 |  |
| Declined to answer | 1 | 0.7 |  |
| *Relationship to Adolescent* |  |  |  |
| Mother | 125 | 92.6 |  |
| Father | 9 | 6.7 |  |
| Aunt | 1 | 0.7 |  |
| *Race* |  |  |  |
| White | 118 | 87.4 |  |
| African American/Black | 17 | 12.6 |  |
| Alaska Native/American Indian | 1 | 0.7 |  |
| Asian Indian | 2 | 1.5 |  |
| Vietnamese | 1 | 0.7 |  |
| Korean | 1 | 0.7 |  |
| Japanese | 1 | 0.7 |  |
| Other race | 2 | 1.5 |  |
| Multiracial | 8 | 5.9 |  |
| *Ethnicity* |  |  |  |
| Not Hispanic/Latino/Latinx/Spanish | 134 | 99.3 |  |
| Hispanic/Latino/Latinx/Spanish | 1 | 0.7 |  |
| *Marital Status* |  |  |  |
| Married | 100 | 74.1 |  |
| Living together | 4 | 3.0 |  |
| Separated | 5 | 3.7 |  |
| Divorced | 12 | 8.9 |  |
| Single | 14 | 10.4 |  |
| *Parent Education Level* |  |  |  |
| High school graduate/GED | 6 | 4.4 |  |
| Some college or training | 8 | 5.9 |  |
| Associate’s degree | 13 | 9.6 |  |
| Bachelor’s degree | 37 | 27.4 |  |
| Graduate/professional degree | 71 | 52.6 |  |

**Table S2.** *Prior-Day Effect on Adolescent Impulsivity*

|  | *Model 1*  *Family Conflict (A)* | *Model 2*  *PC Conflict (A)* | *Model 3  Peer Rejection (A)* |
| --- | --- | --- | --- |
|  | *Est/Var (SE)* | *Est/Var (SE)* | *Est/Var (SE)* |
| Intercept | 2.07 (2.07) | 2.47 (2.12) | 2.51 (1.89) |
| *Within-Person Fixed Effects* |  |  |  |
| Prior-Day Fam Conflict (A) | **0.06 (0.03) *** | ---- | ---- |
| Prior-Day PC Conflict (A) | ---- | **0.05 (0.03)^†^** | ---- |
| Prior-Day Peer Reject. (A) | ---- | ---- | **0.07 (0.03)^††^** |
| Time | **-0.02 (0.00) **** | **-0.02 (0.00) **** | **-0.02 (0.00) **** |
| *Between-Person Fixed Effects* |  |  |  |
| Avg. Fam Conflict | **0.36 (0.14) **** | ---- | ---- |
| Avg. PC Conflict | ---- | **0.38 (0.14) **** | ---- |
| Avg. Peer Reject. | ---- | ---- | **0.54 (0.15) **** |
| Youth Age | 0.02 (0.13) | -0.01 (0.13) | -0.01 (0.12) |
| Youth Sex | 0.26 (0.29) | 0.30 (0.29) | 0.03 (0.27) |
| Family Income | -0.06 (0.05) | -0.05 (0.05) | -0.05 (0.04) |
| *Random Effects* |  |  |  |
| Intercept 𝜎 | 2.45 (1.57) | 2.47 (1.57) | 2.12 (1.46) |
| Daily Family Conflict | 0.01 (0.11) | ---- | ---- |
| Daily PC Conflict | ---- | 0.02 (0.15) | ---- |
| Daily Peer Rejection | ---- | ---- | 1.73 (1.31) |

***Note*:** A = Adolescent-report, PC = Parent-child, Avg. = Average, **p < .01, *p < .05, **^†^**p = 0.078, **^††^** p = 0.060.

**Table S3.** *Prior-Day Effect of Adolescent Impulsivity on Relationships*

|  | *Model 1*  *Predicting Family Conflict (A)* | *Model 2*  *Predicting*  *PC Conflict (A)* | *Model 3   Predicting*  *Peer Rejection (A)* |
| --- | --- | --- | --- |
|  | *Est/Var (SE)* | *Est/Var (SE)* | *Est/Var (SE)* |
| Intercept | **7.48 (2.44) **** | **2.92 (1.41) *** | 1.28 (1.05) |
| *Within-Person Fixed Effects* |  |  |  |
| Prior-Day Impulsivity | -0.01 (0.02) | 0.04 (0.03) | 0.00 (0.02) |
| Time | **0.01 (0.00) **** | -0.01 (0.01) | **-0.01 (0.00) *** |
| *Between-Person Fixed Effects* |  |  |  |
| Avg. Impulsivity | **-0.23 (0.10) *** | **0.15 (0.06) **** | **0.16 (0.04) **** |
| Youth Age | -0.06 (0.15) | -0.09 (0.09) | -0.01 (0.07) |
| Youth Sex | **0.75 (0.34) *** | -0.24 (0.20) | -0.20 (0.15) |
| Family Income | 0.02 (0.06) | -0.04 (0.03) | -0.03 (0.02) |
| *Random Effects* |  |  |  |
| Intercept 𝜎 | 3.55 (1.88) | 1.01 (1.01) | 0.58 (0.76) |
| Daily Impulsivity | 0.00 (1.29) | 0.02 (0.14) | 0.00 (0.02) |

***Note*:** A = Adolescent-report, PC = Parent-child, Avg. = Average, **p < .01, *p < .05.

Level 1 (day-level variables):

(1)$\text{Impulsivity}_{it}=\beta_{0i}+\beta_{1i} \text{day's }\text{Cohesion}_{it}+\beta_{2i} \text{day's }\text{Conflict}_{it}+\beta_{3i} \text{day's Cohesion}\times\text{Conflict}_{it}+\beta_{4i} \text{Time}_{it}+e_{it}$

Level 2 (person-level variables):

$$\left( 2a \right)\beta_{0i}=\gamma_{00}+\gamma_{01} \text{Avg}\text{Cohesion}_{i}+\gamma_{02} \text{Avg}\text{Conflict}_{i}+\gamma_{03} \text{Age}_{i}+\gamma_{04} \text{Sex}_{i}+\gamma_{05} \text{Income}_{i}+u_{0i}$$

$\left( 2b \right)\beta_{1i}=\gamma_{10}+u_{1i}$

${\left( 2c \right) \beta}_{2i}= \gamma_{20}$ $+ u_{2i}$

${\left( 2d \right) \beta}_{3i}= \gamma_{30}$

${\left( 2e \right) \beta}_{4i}= \gamma_{40}$

**Figure 1.** *Multilevel Model Formula for Daily Impulsivity*

***Note.*** This figure presents the Level 1 (day-level) and Level 2 (person-level) equations used to model adolescents’ daily impulsivity as a function of daily family cohesion and conflict.

In level 1, ${\text{i}\text{mpulsivity}}_{it}$ reflects adolescent impulsivity on day $t$ for person $i$; $\beta_{0i}$ represents the expected impulsivity, adjusted for between-person predictors, on an average day in the middle of the study; $\beta_{1i}$​ and $\beta_{2i}$​ represent the associations between day's family cohesion and conflict and impulsivity, respectively; $\beta_{3i}$​ captures their interaction; and $\beta_{4i}$​ indicates the association between study time and adolescents’ daily impulsivity. Day-specific residuals ($e_{it}$) were allowed to autocorrelate (AR1).

In level 2, the $\gamma$ terms represent fixed effects at the sample level, and the $u$ terms represent person-level residual variation. As shown in Equations 2a–2e, between-person predictors (average cohesion, conflict, age, sex, and income) explain variability in adolescents’ average levels of impulsivity. Random slopes were estimated for day-level cohesion and conflict.
